# Ten simple rules for launching an academic research career

**DOI:** 10.1371/journal.pcbi.1010689

**Published:** 2022-12-15

**Authors:** Jason A. Papin, Jessica Keim-Malpass, Sana Syed

**Affiliations:** 1 Department of Biomedical Engineering, School of Medicine, School of Engineering & Applied Sciences, Charlottesville, Virginia, United States of America; 2 Department of Biochemistry & Molecular Genetics, School of Medicine, Charlottesville, Virginia, United States of America; 3 Division of Infectious Diseases & International Health, Department of Medicine, School of Medicine, Charlottesville, Virginia, United States of America; 4 Department of Acute and Specialty Care, School of Nursing, Charlottesville, Virginia, United States of America; 5 Department of Pediatrics, School of Medicine, University of Virginia, Charlottesville, Virginia, United States of America

At the beginning of an independent academic research career as a principal investigator of a lab or similar professional position, the future can seem like a looming set of overwhelming hurdles. As soon as possible, there is a need to start assembling grant applications, establish the experimental or computational infrastructure to generate the data for those manuscripts and grants, recruit staff and graduate students to grow the research activity…where does someone even begin?!?

Here, we share *Ten Simple Rules* in an attempt to suggest how to make sense of things as someone launches their academic research career. We share these ideas from the perspective of our own academic research careers as well as our experience leading or participating in an NIH-funded research career development program (https://www.ithriv.org/ithrivscholars-program-main).

We appreciate there are an infinite number of unique paths towards many different kinds of “successful” academic research careers [1] and that there are different time points at which an individual may enter or transition into an independent research position. Our suggestions only represent lessons from our own experiences. With the perspective of our own diverse backgrounds, we aim to share principles that we have found to be important for our research career development. We recognize that privilege can play an enormous role as an individual navigates a career path; academic researchers who are first-generation college students, who have varied parenting or elder care responsibilities, or who have lived experiences from the perspective of a variety of racial or ethnic minority backgrounds or members of the LGBTQ+ community will all have important additions, subtractions, or modifications to these proposed rules [2–5]. We encourage all readers to reach out to whatever mentors or resources they can find that will add nuance necessary for the implementation of these proposed rules in their unique circumstance.

It is day 1 of your independent academic research career. Now what?

## Rule 1: Don’t be afraid of the empty space…just take a step inside, even if it’s the wrong one

For nearly all academic researchers newly stepping into an independent position, this will be the first time to actually start from scratch. In comparison to your time as a trainee where there was often significant infrastructure in place, all the equipment, supplies, workflows, staff …everything… will need to be purchased/organized/initiated/recruited by you. This may seem like an Everest-like task requiring a herculean effort. But it has been done by many, and you can absolutely be successful at getting a functional lab up and running…you were hired because a committee of people had faith that you were capable of independently establishing a lab and directing it from scratch! The first step may seem like the hardest, but you just have to start in order to actually get there. So, place that order! Talk to that prospective graduate student! Talk to the human resources department to get that job position posted! Take at least 1 step in the direction you want to go, and you will find it easier to make those executive decisions and begin to realize the vision you started with. Remember that risk-taking in science often leads to new methodological perspectives. So, do not fear the empty space. Look at it as a creative space and approach it as an artist regarding a blank canvas brimming with ideas. Just take that first step.

## Rule 2: Reach out to scientific and professional mentors

Someone has likely experienced the same challenges and questions you have. It can be intimidating to admit your vulnerability to the professional colleagues around you, to admit that you are confronting a challenge or question and simply do not know what to do next. But, if you stop to think about it, you are certainly not the first to face a question of how to deal with some kind of institutional bureaucracy, or how to successfully attract that student to work in your group, or how to respond to critiques of a grant proposal. Also, our experience is that for a vast majority of the time, senior academic researchers actually want to help. It is flattering for a more junior colleague to approach a more senior colleague with a question, seeking insight for which the senior colleague’s opinion is valuable. If a more senior colleague wants to help, why not ask? Perhaps there is fear that the more senior colleague will think “less” of the junior colleague. From our experience, this is rarely the case, and if so, that individual is probably not the kind of colleague with whom you would want to nurture a mentoring relationship in the first place. In addition, keep in mind that different kinds of mentors and sponsors can support you for different reasons. One mentor may offer incredible insight on how to draft responses to the critiques of a grant application. Another mentor may be particularly adept at helping to navigate institutional bureaucracy. Yet another mentor consistently reaches out to you about opportunities for recognition and is willing to sponsor you. Reach out to all those with expertise around you. In addition, there are several online communities that provide excellent advice on all kinds of facets of launching a research career [6,7]. One more note on the constellation of mentors you build around yourself; sometimes the most effective mentors for specific needs will be the administrative support staff with whom you frequently interact. While research mentors are essential, you need support staff to stand next to you. So, leverage the infrastructure, treat staff with respect, and ask for help!

## Rule 3: Find a peer cohort

We all need a little help from our friends. There is power in knowing you are not alone as you try to figure out how to navigate your fledgling academic research career. There is much literature on the positive value of a cohort of peer colleagues with whom you feel like you can face the various challenges together [8–10]. There is value in simply knowing you are not alone. Perhaps it is a weekly lunch with a handful of other researchers at your institution. Perhaps it is a texting/Slack/email group where you can simply share experiences/frustrations/advice. Help create your community so you and they can support one another. Again, remember that there are benefits to having members of your cohort from a variety of disciplines and departments. We learn best with a diverse group of people around us, and other newly independent investigators around you can offer fresh perspectives on approaching the challenges all are facing. Remember, this peer cohort can be composed of faculty who are perhaps a couple years further along their career paths than you are—they will often be willing to share lessons learned from experiences more recent than those of your more “senior” colleagues and your outreach will help them feel valued.

## Rule 4: Learn the inside of institutional processes (IRBs, IACUC, proposal submission, budgeting, expense tracking, hiring of staff), without getting bogged down in the minutiae

Nearly all independent academic career positions will exist within some kind of institutional structures that inherently have processes in place to support grant applications, student training, staff support, among other things. Remember, these structures are often in place to ensure compliance with government agencies; they are there to help. It is important to learn how your institution executes these various functions so that your own research activities can be supported. However, it is possible to get so bogged down in all the minutiae of these processes that you never actually complete the task that you want to do. As long as you move forward with good faith, people will correct you when you have done something wrong and then you can work through the process even better the next time. An attitude of “I will make a mistake, but I am trusting experienced institutional processes to help point out my blind spots,” will go a long way to establishing you as a researcher to trust and respect.

## Rule 5: Learn to manage people within your group

Individuals launching an academic research career rarely receive formal training for managing trainees and support staff. Yet that is one of the things that they are most responsible for doing from the very beginning. Take advantage of workshops at your institution on management best practices. Look to resources within this journal on communicating clear mentor–mentee expectations [11], running productive lab meetings [12], and creating a healthy lab culture [13]. In addition, there are many books and resources on management practices that can help guide your efforts [14–17]. Take advantage of training in leadership practices when you are able. Such leadership training resources may be available through your institution or professional societies. The important thing is to be deliberate and to learn leadership skills as you can along the way. The trainees and support staff that you are responsible for each have their own goals and aspirations, communication styles, and scientific strengths and weaknesses. Your responsibility is to make the effort to manage and support with the best practices you can so that the group as a whole can move forward to achieve its research goals.

## Rule 6: Learn to manage people outside your group

An independent academic research position also involves managing individuals outside your own research group. Collaborators, institutional colleagues, and relationships with community organizations and individuals outside academia can enrich many aspects of the job, but it is essential in all these relationships to communicate expectations and learn communication preferences. A good collaborator can elevate everything about the job. A difficult collaborative relationship can negatively affect all aspects about the job. Most collaborators and colleagues will have good intentions; your job is to learn how to best manage those relationships so all can benefit. Learn from best practices shared by others for successful management of collaborations [18]. Formal training (see Rule #5) in academic leadership can also help you create your own set of tools to manage these relationships.

## Rule 7: Be vulnerable with your ideas

The easiest way to develop bad ideas is to never share them. Science often works best through peer review, and the best time to get peer review is before submitting that proposal/manuscript/abstract so that you can restructure what you are presenting so that it is compelling to whatever anonymous reviewer evaluates it. Do not be afraid to be vulnerable with your ideas to those you trust. Again, you were hired into the position in the first place because a committee of colleagues believed in your ability to generate new scientific ideas, craft a plan to address them, and subsequently how to present them to the larger community. Share whenever you can through grant-brewing sessions, journal clubs, Research-in-Progress talks; share your specific aims, presentation drafts, abstract drafts, papers-in-progress as you are able and with colleagues that can give honest feedback. It will only make the products you generate even better. Remember to not let perfect be the enemy of excellent enough; even if you think your proposal/paper/abstract is flawless, chances are the reviewers will have a different opinion and feedback you receive can help improve your future work.

## Rule 8: Consider diversity from the beginning and throughout

There is extensive evidence of the value added by mentors, trainees, and others with diverse perspectives and lived experiences [19]. As you develop a cadre of mentors and as you recruit trainees and other staff, it is critical to be deliberate in considering diversity from the beginning. As these diverse perspectives are integrated, your group will be more creative and have even greater scientific impact. Diversity should be considered in how lab expectations are developed and communicated, as well as how performance is measured and evaluated [20]. Trainees graduate, staff move on to other positions, and mentors will move on to other institutions. You will again be in the role of recruiting individuals as mentors or to support the lab’s activity. Be sure to consistently make the effort to consider diversity as much as possible along the way—several federal agencies have provided extensive data that can help increase your awareness of diversity challenges [21,22]. Remember that what makes an individual “diverse” may also make it difficult to easily recruit them. Take advantage of institutional outreach strategies, community groups, and other organizations that can amplify your recruiting efforts to reach these individuals.

## Rule 9: Balance diversifying your research activity with maintaining focus on your core research expertise

It can be dangerous to depend on that one grant application that you think must get funded or that one paper that just has to be published. It is impossible to have complete control over the successes of every aspect of your research career; sometimes that one “perfect” grant application is just not getting traction and then you are stuck with how you support your lab’s research activity. It is necessary to have a few projects that you nurture along the way. Our experience is that sometimes the project that really resonates with a set of reviewers may not be the one you anticipate. While you work to diversify your research activity, keep in mind that too many disparate projects increasingly become too difficult to manage: too much varied literature to follow or perhaps too much different expertise that each member of your group needs to independently develop [23]. It can be difficult to develop a reputation for a specific expertise if your published work is all over the place. Diversify your research activity in strategic ways, taking the core set of expertise in important new directions that allows your research group to build on what it knows. Strategic diversification allows you to also build and add to your methodological toolbox and master new skills. Finally, acknowledge the power of team science in diversifying your portfolio. Often, adding co-investigators with varied backgrounds can diversify your research while maintaining your core focus.

## Rule 10: Solicit and integrate feedback (just not too much of it)

As stated above, the feedback you solicit from mentors and that you will receive through various review processes is essential to improve your science. However, it is common to receive conflicting feedback that becomes impossible to reconcile or so much feedback that it becomes difficult to sufficiently address everything and still meet deadlines that you confront. Remember that you are the world’s expert on your science. Only you have the vision to truly craft your personal career path. Furthermore, there are certainly additional demands on your time and focus (e.g., teaching, service, administration, family care, self-care) that will have to be balanced and need to be considered as you adapt to the feedback and advice you receive. It is important to take the feedback and then make a thoughtful judgment about which items are most important to consider.

We have found an academic research career to be an incredibly rewarding experience with frequent opportunities to be creative and work with amazing people. These Ten Simple Rules are lessons that we have learned along an oft winding road (sometimes in difficult ways!) and witnessed in the academic research careers of many colleagues along the way. We also encourage the reader to explore other Ten Simple Rules that highlight important aspects of a research career, including serving as the principal investigator of a research program [24], approaching a new job [25], and considering a career in academia versus government [26]. As an academic research career is launched, we have also found immense satisfaction in the overall mission of discovery and training that is central to all that we do.
